# Effects of health qigong exercise on upper extremity muscle activity, balance function, and quality of life in stroke patients

**DOI:** 10.3389/fnins.2023.1208554

**Published:** 2023-07-19

**Authors:** Huixin Yang, Baolong Li, Lin Feng, Zhonglou Zhang, Xiaolei Liu

**Affiliations:** ^1^Institute of Nation Traditional Sport, Harbin Sport University, Harbin, China; ^2^The Second Affiliated Hospital of Heilongjiang University of Traditional Chinese Medicine, Harbin, China; ^3^Chinese Traditional Regimen Exercise Intervention Research Center, Beijing Sport University, Beijing, China

**Keywords:** Qigong, upper extremity, muscle activity, balance function, quality of life, stroke patients

## Abstract

**Introduction:**

This study explored the effects of Qigong exercises on upper extremity muscle activity, balance function, and quality of life in stroke patients.

**Methods:**

A total of 30 stroke patients were randomly allocated to either control group or Qigong group. In the Qigong group, participants completed an intervention of Qigong Baduanjin over 8 weeks. Data on the electromyographic activities of the biceps brachii muscle, triceps brachii muscle, and muscle coordination were obtained using surface electromyography and the co-contraction ratio (CCR). Data on balance were obtained using the PK254P balance function detection system. Quality of life was measured using the brief version of the World Health Organization Quality of Life scale.

**Results:**

The results for the Qigong group showed a significant difference in CCR of the triceps brachii muscle (*p* < 0.01). Concerning balance (assessed using the open-eye test), there was a significant decrease (*p* < 0.05) in Y-axis trajectory deviations and the Y-axis speed in the Qigong group. In the closed-eye test, the peripheral area of the Qigong group was significantly lower than that of the control group (*p* < 0.05). Significant differences were also observed in physical health (*p* < 0.05), psychological health (*p* < 0.01), environment (*p* < 0.01), and the total scores for quality of life (*p* < 0.01) in the Qigong group.

**Discussion:**

We conclude that Qigong exercises improve the quality of life in stroke patients and have positive effects on the coordination of limb extremities and balance function.

## 1. Introduction

According to a bibliometric analysis of the clinical studies on Qigong, the top 15 most commonly studied diseases/conditions are diabetes, chronic obstructive pulmonary disease, hypertension, stroke, cervical spondylosis, lumbar disc herniation, insomnia, knee osteoarthritis, lower back pain, osteoporosis, coronary heart disease, breast cancer, periarthritis of the shoulder, depression, and metabolic syndrome. Thus, considerable research has shed light on the effects of Qigong following a stroke (Zhang et al., 2020).

Stroke is the second leading cause of death (after cancer) globally (Feigin et al., 2017), accounting for 11.8% of all-cause mortality (Valery et al., 2018), and there are roughly 17 million new cases each year (Crichton et al., 2016). Once the sensory or motor conduction in stroke patients is impaired, they may lose muscle strength or control, partly or wholly (Vahlberg et al., 2013, 2017). Such impairments can impede patients’ daily activities and lead to long-term weakness, paresis, or falls (Taylor-Piliae et al., 2014; Pereira et al., 2019).

In a recent study, a 10-day mind–body interactive exercise program, Chan-Chuang qigong, was shown to benefit subacute stroke inpatients and offer useful adjunctive rehabilitative care for stroke inpatients. Among the covariates in this study, neurologic deficit, muscle strength, low-frequency to high-frequency ratio, and anxiety were significantly associated with changes in quality of life (Chen et al., 2019). A longer, 12-week supervised Baduanjin exercise intervention was effective and safe in modulating cerebral hemodynamics, reducing blood pressure, and improving anthropometric parameters and related psychological outcomes in older community-based adults at risk for ischemic stroke. After the 12-week intervention period and additional 12-week follow-up period, the Baduanjin exercise group displayed significant changes in most cerebral hemodynamic parameters compared to the control group: lower systolic blood pressure, diastolic blood pressure, plasma total cholesterol levels, waist circumference, hip circumference, and waist/hip ratio; and improved mood, self-confidence, self-esteem, quality of life, and sleep quality (Zheng et al., 2019). Meanwhile, regular Baduanjin training has been associated with less loss of cognitive function in patients after stroke. Compared to the control group, Baduanjin training significantly increased Montreal Cognitive Assessment scores for global cognitive function from 16 weeks after the intervention. It also improved scores for immediate recall and short-term delayed recall from 16 weeks after the intervention, and long-term delayed recognition scores at 24 weeks after the intervention in the memory domain. In addition, it shortened the time taken to complete the Trail Making Test-A and Trail Making Test-B tests from 16 weeks after the intervention in the executive functioning domain. These effects were maintained from 4 weeks until after follow-up (i.e., 28 weeks after the intervention). In the mixed linear model analyses, significant main effects or interaction effects for exercise were found for those measures (Zheng et al., 2020).

Research has examined whether Qigong exercises can improve physical and mental health indices such as sleep quality, gait performance, and muscle strength (Wang et al., 2014; Wu et al., 2016; Klich and Milert, 2018; Moreno-Segura et al., 2018). In stroke patients, the stability of the core muscle group is weak, and the trunk and pelvis cannot be stabilized during anti-gravity activities, which may weaken the motor and balance function in patients (Feigin et al., 2014; Valery et al., 2018). As a low-intensity activity, Qigong exercises can increase patients’ muscle flexibility and strength, thus alleviating muscle tension and improving balance, mental health, and quality of life (Feigin et al., 2015; Crichton et al., 2016). Therefore, Qigong exercises have been used to improve balance (Taylor-Piliae et al., 2014; Xu et al., 2017) and endurance (Xu et al., 2017) in the rehabilitation of stroke patients. However, there are few studies about the positive effects of Qigong on stroke patients with moderate impairments of upper limb strength and function, and the relationship between upper limb extremity muscle activities and balance function.

In this study, we evaluated Qigong exercises on stroke patients’ upper extremity muscle activity, balance function, and quality of life, regarding the benefits of Qigong. It showed the data of the stroke patients before and after the experiment to see how Qigong works on the physical health, like the muscles and joints, and mental health, like the quality of life.

## 2. Materials and methods

### 2.1. Design

**Figure 1:**
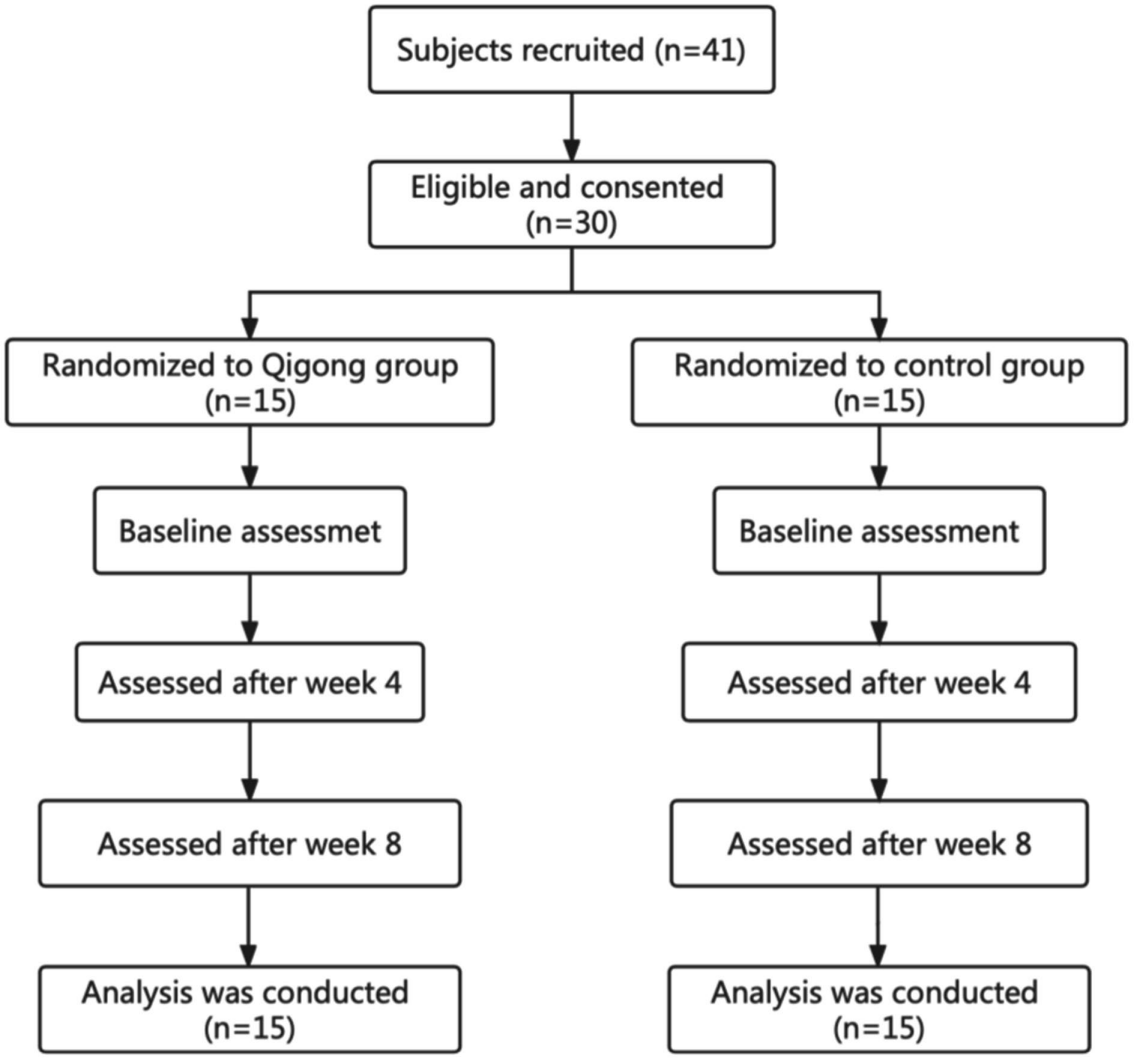
The flowchart of the experiment.

This study was based on an 8-week randomized controlled design with two groups (a control group and a Qigong group). The data were collected at the baseline, after 4 weeks and at the end of week 8 (Figure 1). Subjects in the Qigong group practiced Qigong Baduanjin while the control group subjects did not receive or perform any regular physical activity. The study protocol was approved by the Internal Review Board of the Heilongjiang University of Chinese Medicine. All eligible subjects signed an informed consent form before the study. Data from all the subjects admitted to the hospital consecutively were collected with clinical registration (Registry Number: ChiCTR2100048031) and ethical approval (Approval Number:2021-K135) by the Ethics Committee of the Second Affiliated Hospital of the Heilongjiang University of Chinese Medicine.

### 2.2. Subjects

The sample size was ascertained using G-power before the experiment. By setting the effect size to 0.25, power to 0.8 and alpha to 0.05, we can get 29 as the minimum of the total number of the subjects. In addition, considering 15% attrition rate, at least 35 subjects should be recruited at the very beginning. Therefore, a total of 41 patients with a clinical diagnosis of stroke were voluntarily recruited from the Second Affiliated Hospital of the Heilongjiang University of Chinese Medicine. They were in Brunnstrom stages IV–V. The inclusion criteria for patients were as follows: (1) aged less than 75 years; (2) had experienced their first, officially diagnosed stroke; (3) were at the stage of motor recovery (Brunnstrom IV-V stage); (4) experienced the onset of stroke more than 1 week but no more than 3 months ago; (5) were able to walk more than 6 meters freely without any support; (6) had a Mini-Mental State Exam score higher than 24. Patients were excluded if they had (1) a hearing impairment, a neurological impairment, or sensory aphasia that influenced training, or (2) severe comorbidities (e.g., diabetes, heart disease, cancer, brain tumor). A total of 11 patients withdrew from the study, meaning that 30 patients were studied and the requirements of the G-power calculation were met.

All the information regarding the intervention was concealed from the subjects and the testers, but not the researchers, ensuring the study was blinded. Subjects did not know which group they have been placed in until the experiment finished. As the control group, they were treated with the same Qigong methods after the experiment. The subjects were randomly allocated using random, computer-generated numbers into the Qigong group or the control group such that there were 15 subjects per group. An independent investigator generated the random numbers and was not involved in the intervention and assessment.

### 2.3. Intervention

The control group did not receive any specific exercise training and these participants were asked to maintain their routine medical or rehabilitative treatment and original physical activity. They were able to avail themselves of the Qigong exercises after the study.

The Qigong group completed the Baduanjin exercises 3 days a week for 40 min a day based on their routine medical or rehabilitative treatment at the Heilongjiang University of Chinese Medicine. The Qigong master had a 10-years teaching experience. Each session consisted of 5–10 min warm-up and cool-down routines that focused on stretching, rhythmic breathing, and relaxation followed by 30 min of Qigong practice (including movements and repetitions). All the subjects gathered together to train as part of the same community and were guided by qualified Baduanjin exercise coaches. The whole process for the Qigong group was video-recorded so patients could practice in their own time.

### 2.4. Measurements and procedure

Subject characteristics such as age, gender, duration of disease, and education were collected via a questionnaire at the beginning of the study. Four instruments were used as measurement tools. The muscle activity of the biceps brachii and triceps brachii was assessed using sEMG (Thought Technology, SA7550, Canada) and the Ag/AgCI electrode (CH55D) with an inter-electrode distance of 2 cm. These data were collected at baseline, after 4 weeks and at the end of week 8.

Surface electromyography (sEMG) is an inexpensive, quantitative, non-invasive, and painless method to evaluate neuromuscular function (Ford et al., 2008). In the mid-twentieth century, sEMG was used to recognize neuromuscular patterns, identify the roles of different muscles in functional movements, and determine prognosis following nerve injury (Vahlberg et al., 2013, 2017). In the following decades, sEMG was used to examine neurological factors such as the coordination between limbs, patterns of muscle activation and co-activation, and biofeedback effects. More recently, sEMG has been used to identify and treat gait disorders in stroke survivors (Liu et al., 2011; Pollock et al., 2014; Pereira et al., 2019).

The PK254P balance function detection system (Wu and Gong, 2019) is used to test the balance of patients when standing upright with their eyes opened and closed. In this study, the PK254P was used to collect data from two groups, the control group and Qigong group. The indexes included the trajectory circumference (TL), the peripheral area (Area), the X-axis trajectory deviations (X dev.), and the Y-axis trajectory deviations (Y dev.). The indexes indicate the stability of the center of gravity in stroke patients. The X-axis speed (X speed) and the Y-axis speed (Y speed) show the micro-control of the center of gravity. X and Y axes are the speed indices of PK254P. X axis represents the velocity in the left and right direction. Y axis represents the velocity in the anteroposterior direction. In addition, the World Health Organization Quality of Life-Brief version (WHOQOL-BREF) scale was used to test each patient’s quality of life.

Before testing, each patient sat on the testing chair with their knees bent so that their lower legs were at 90 degrees to their thighs and the floor. Testers shaved the skin on each patient’s upper limbs and swabbed the area with alcohol before attaching the electrodes. Electrodes were attached to the biceps brachii and triceps brachii muscles. Patients were familiar with the function of the electrodes and understood the instructions that followed. The temperature of the test room was kept between 22 and 28°C.

During testing, the sEMG signals were collected in five-second segments and the measurement for each position (at elbow extension and elbow flexion) was repeated five times, following the system prompt. The sEMG results of the biceps brachii and triceps brachii muscles in the intermediate three activities were recorded, as per the method used by Zhu et al. (2014).

The co-contraction ratio (CCR) represented the ratio of normalized agonistic muscle activity to normalized antagonistic muscle activity during the intervention. A decreasing CCR value signified a decrease in antagonist co-activation, higher coordination, and smoother movement (Lim et al., 2016). CCR was calculated according to the formula reported by Hammond et al. (1988) as follows:


$$\mathrm{CCR}=\frac{sEMG\left(antagonisticmuscle\right)}{sEMG\left(agonisticmuscle\right)+sEMG\left(antagonisticmuscle\right)}$$

Using the Pk254P balance function detection system, the TL, Area, X dev., Y dev., X speed, and Y were selected as the speed indexes in each group. The smaller these indexes, the better the individual’s balance ability.

The WHOQOL-BREF was used to assess patients’ quality of life and included an assessment of each participant’s physical, mental, and social states, and their environment before the intervention, then 4 and 8 weeks after the intervention. All assessments were completed by the same tester who had been trained for the work and did not know of the difference between the two groups.

### 2.5. Statistical analysis

The software SPSS 17.0 (SPSS Inc., Chicago, United States) was used for statistical analysis, and a two-sided *p* < 0.05 was set to determine statistical significance. Measurement data are expressed as means and standard deviations (*SD*). The Kolmogorov–Smirnov test was used to test the normality of distributions and the chi-squared test was used to compare the categorical variables for the two groups at baseline. The Kruskal-Wallis test and one-way analysis of variance (ANOVA) were used to analyze the categorical variables and continuous variables at baseline and across groups, respectively. Comparison between groups was performed using a mix-model repeated ANOVA (group × time) while comparisons within each group were conducted using paired *t*-tests.

## 3. Results

### 3.1. Characteristics of study participants

Of the 30 participants, 15 were in the control group, and 15 were in the Qigong group. Table 1 shows the detailed characteristics of the participants at baseline. At baseline, there were no significant differences between the groups for the continuous variables (age and duration of disease). The chi-squared test for categorical variables (gender, lesion nature, Brunnstrom stage, and education) also showed no significant differences between the groups at baseline. Furthermore, there was no significant difference in the bias of the impairments, especially the postural tone, which is also listed in Table 1.

**Table 1:** Baseline characteristics of two groups (*n* = 30).

Variable		Control group (*n* = 15)	Qigong group (*n* = 15)	*p-*value
Age (year)		54.02 ± 38.41	52.85 ± 32.85	0.88
Length of disease (day)		57.57 ± 60.34	52.57 ± 56.12	0.77
Gender	Male	10	11	0.28
	Female	5	4	0.91
Lesions nature	Cerebral infarction	11	10	0.83
	Encephalorrhagia	4	5	0.72
Brunnstrom stage	IV	4	7	0.58
	V	11	9	0.58
Postural tone	High	4	3	0.99
	Low	11	12	

### 3.2. Effects of Qigong exercises on upper extremity muscle activity

#### 3.2.1. sEMG results of biceps brachii muscle and triceps brachii muscle

As shown in Table 2, there was a significant main effect of time for the biceps brachii (elbow extension). No significant main or interaction effects were found for biceps brachii (elbow flexion) and triceps brachii (elbow extension and elbow flexion).

**Table 2:** Descriptive statistics of all outcome variables across groups at baseline and post-intervention.

	Qigong group			Control group			Group	Time	Interaction effect
	V0	V1	V2	V0	V1	V2	F/P	F/P	F/P
Biceps brachii (elbow extension)	41.13 ± 13.48	36.08 ± 15.05	29.99 ± 11.29	42.39 ± 17.96	37.39 ± 15.87	30.64 ± 9.78	0.156/0.697	3.327/0.047	0.003/0.996
Triceps brachii (elbow flexion)	93.45 ± 25.56	96.17 ± 23.29	97.08 ± 21.54	91.87 ± 30.49	98.31 ± 32.72	100.08 ± 23.67	0.062/0.806	0.275/0.747	0.042/0.951
CCR of triceps brachii	0.30 ± 0.06	0.27 ± 0.07	0.23 ± 0.06**	0.31 ± 0.07	0.28 ± 0.06	0.24 ± 0.05*	0.341/0.565	7.570/0.002	0.038/0.961
Triceps brachii (elbow extension)	31.80 ± 9.74	30.75 ± 10.09	28.12 ± 10.56	29.15 ± 13.47	28.08 ± 9.69	27.64 ± 12.35	1.065/0.313	0.269/0.756	0.063/0.933
Biceps brachii (elbow flexion)	95.12 ± 26.33	100.64 ± 25.50	109.02 ± 31.79	92.99 ± 26.59	98.94 ± 26.48	102.58 ± 26.18	0.288/0.597	1.048/0.356	0.052/0.941
CCR of biceps brachii	0.26 ± 0.06	0.23 ± 0.06	0.20 ± 0.04	0.24 ± 0.11	0.22 ± 0.07	0.21 ± 0.07	0.068/0.797	1.869/0.176	0.144/0.816

V0, V1, V2 indicated pre-test, after 4-week intervention, after 8-week intervention, respectively. *Represented for *p* < 0.05; **represented for *p* < 0.01.

#### 3.2.2. CCR of biceps brachii and triceps brachii

As shown in Table 2, main effects of time were found for the CCR of triceps brachii (*p* < 0.01), in which subjects’ scores significantly decreased from baseline to week 8, regardless of group allocation. After week 8, the CCR of triceps brachii in the Qigong group changed by 0.07 ± 0.07 (*p =* 0.008), which indicated a significant improvement compared with pre-test.

### 3.3. Effects of Qigong exercises on balance function

As shown in Table 3, in the open-eye test, a main effect of time was found for Y dev. (*p* < 0.05), reflecting improvements in both the Qigong group and the control group after 8 weeks. In the Qigong group, the scores of the Y speed and Area significantly decreased (*p* < 0.05 and *p* < 0.01, respectively) after the 8-week intervention.

**Table 3:** Comparison of static balance indexes in the open-eye of biped (X ± S).

	Qigong group			Control group			Group effect	Time effect	Interaction effect
	V0	V1	V2	V0	V1	V2	F/P	F/P	F/P
X dev. (mm)	7.00 ± 1.76	6.87 ± 2.36	6.09 ± 1.83	7.48 ± 2.23	6.95 ± 1.94	6.62 ± 2.18	0.803/0.375	2.417/0.100	0.181/0.817
Y dev. (mm)	7.74 ± 1.74	7.52 ± 2.15	6.57 ± 1.95*	7.67 ± 2.20	7.24 ± 1.84	6.43 ± 1.69*	0.230/0.634	4.616/0.013	0.035/0.966
X speed (mm/s)	11.09 ± 2.45	10.61 ± 2.84	10.30 ± 1.55	11.43 ± 2.82	10.43 ± 3.25	10.33 ± 1.62	0.024/0.876	1.619/0.208	0.114/0.859
Y speed (mm/s)	11.87 ± 3.07	11.65 ± 2.21	10.35 ± 1.82*	11.57 ± 3.22	11.33 ± 2.71	10.57 ± 1.78	0.062/0.805	3.971/0.028	0.208/0.782
TL (mm)	524.83 ± 170.49	504.43 ± 172.53	429.26 ± 139.87	529.95 ± 189.99	521.81 ± 199.70	510.43 ± 129.54	1.327/0.256	1.418/0.248	0.658/0.513
Area (mm^2^)	759.65 ± 202.45	686.48 ± 243.47	607.00 ± 133.55**	747.19 ± 227.74	636.62 ± 266.38	612.24 ± 128.13*	0.363/0.550	4.884/0.014	0.182/0.801

V0, V1, V2 indicated pre-test, after 4-week intervention, after 8-week intervention, respectively. *, **V0 compared with V2, *P <* 0.05, *P <* 0.01.

As shown in Table 4, in the closed-eye test, except for the scores of Area, in which the Qigong group showed a significant decrease, no significant improvement was found.

**Table 4:** Comparison of static balance indexes in closed-eye of biped (X ± S).

	Qigong group			Control group			Group effect	Time effect	Interaction effect
	V0	V1	V2	V0	V1	V2	F/P	F/P	F/P
X dev. (mm)	8.96 ± 1.72	8.57 ± 2.13	7.78 ± 1.70	9.24 ± 2.10	9.05 ± 1.83	8.95 ± 2.24	2.531/0.119	1.542/0.220	0.565/0.570
Y dev. (mm)	9.91 ± 2.37	9.61 ± 2.29	8.61 ± 1.88	9.62 ± 2.80	9.48 ± 2.06	8.10 ± 2.05*	0.675/0.416	4.827/0.011	0.076/0.924
X speed (mm/s)	15.43 ± 2.69	15.09 ± 2.94	14.57 ± 2.95	15.52 ± 2.84	14.43 ± 3.25	14.43 ± 2.181	0.001/0.978	0.739/0.477	0.422/0.650
Y speed (mm/s)	15.52 ± 2.97	15.30 ± 3.18	14.22 ± 1.93	14.95 ± 3.12	14.52 ± 3.71	14.52 ± 1.778	0.336/0.565	1.324/0.272	0.573/0.562
TL (mm)	669.48 ± 163.17	693.43 ± 229.03	667.91 ± 211.56	736.81 ± 196.08	721.33 ± 221.88	724.24 ± 196.01	1.455/0.234	0.043/0.952	0.135/0.863
Area (mm^2^)	968.57 ± 294.27	935.3 ± 373.95	771.04 ± 244.46*	935.71 ± 289.66	913.81 ± 326.38	875.19 ± 224.04	0.108/0.744	2.244/0.116	0.703/0.489

V0, V1, V2 indicated pre-test, after 4-week intervention, after 8-week intervention, respectively. *V0 compared with V2, *P* < 0.05, *P* < 0.01.

#### 3.3.1. Effects of Qigong exercises on quality of life

The results showed a significant main effect of time (*p* < 0.001) for the total WHOQOL-BREF score (Table 5). In the Qigong group, significant differences were found at week 8 (*p* < 0.01) as compared with baseline.

**Table 5:** WHOQOL-BREF score comparison (X ± S).

	Qigong group			Control group			Group effect	Time effect	Interaction effect
	V0	V1	V2	V0	V1	V2	F/P	F/P	F/P
Physical health	13.69 ± 2.25	14.21 ± 2.20	15.18 ± 3.00*	13.50 ± 1.97	14.04 ± 1.79	14.82 ± 2.48	0.307/0.583	4.465/0.015	0.025/0.973
Psychological health	12.17 ± 1.65	13.11 ± 2.49	14.87 ± 2.60**	11.97 ± 1.71	12.92 ± 2.06	13.33 ± 2.49*	2.585/0.115	9.776/0.000	1.410/0.250
Social relationships	10.07 ± 3.60	10.96 ± 2.57	12.06 ± 2.97	9.71 ± 3.38	10.41 ± 3.57	10.98 ± 2.79	1.642/0.207	2.725/0.072	0.142/0.868
Environment	13.26 ± 2.15	14.28 ± 2.13	15.02 ± 2.68**	12.98 ± 2.16	13.12 ± 2.24	13.36 ± 2.37	6.851/0.012	2.364/0.101	1.003/0.370
Total	54.36 ± 4.80	56.25 ± 4.83	60.78 ± 5.25**	54.04 ± 6.43	55.25 ± 5.11	58.24 ± 5.40*	1.586/0.215	13.018/0.000	0.564/0.571

V0, V1, V2 indicated pre-test, after 4-week intervention, after 8-week intervention, respectively. *, **V0 compared with V2, *P* < 0.05, *P* < 0.01.

For physical health, there was a significant main effect of time (*p* < 0.05) but no main effect of group or interaction. The score of the Qigong group had significantly decreased at week 8 (*p* < 0.05) and there was a significant difference between the pre-test and post-test (*p* < 0.05).

A main effect of time was also found for psychological health. In the Qigong group, the scores increased significantly at week 8 (*p* < 0.01). At the same time, the control group showed a significant increase (*p* < 0.05) at week 8.

No significant main or interaction effects were found for social relationship scores.

However, for the environment scores, a main effect of group (*p* < 0.05) was found, demonstrating a significant difference between the Qigong group and the control group. The scores for the Qigong group increased continually over the study and showed a significant improvement at week 8 (*p* < 0.01).

## 4. Discussion

Previous studies have suggested that training can improve the balance of the human body and thus enhance balance control (Horak et al., 1997). Studies also found that 4 weeks of intensive practice of Tai Chi improved standing balance in healthy seniors and 12 weeks of short-form Tai Chi produced specific standing balance improvements in people with a chronic stroke that outlasted training for 6 weeks (Au-Yeung et al., 2009). One session of Qigong exercise improved energy and balance between the upper and lower halves of the body (Lin et al., 2018), and the addition of Baduanjin to conventional cardiac rehabilitation exercise further improved exercise endurance, the reserve of heart function, and quality of life (Yu et al., 2018). Another study suggested that Chan-Chuang Qigong, a mind–body interactive exercise, is an appropriate and desirable healthcare intervention for subacute stroke inpatients. The Chan-Chuang Qigong intervention was found to improve the physical and mental aspects of quality of life when considering the severity of stroke impairment, muscle strength, sympathovagal balance, anxiety, and depression, supporting the view that Chan-Chuang Qigong is a simple and safe adjunctive approach for stroke inpatients when practiced for at least 10 days (Chen et al., 2019).

**Figure 2:**
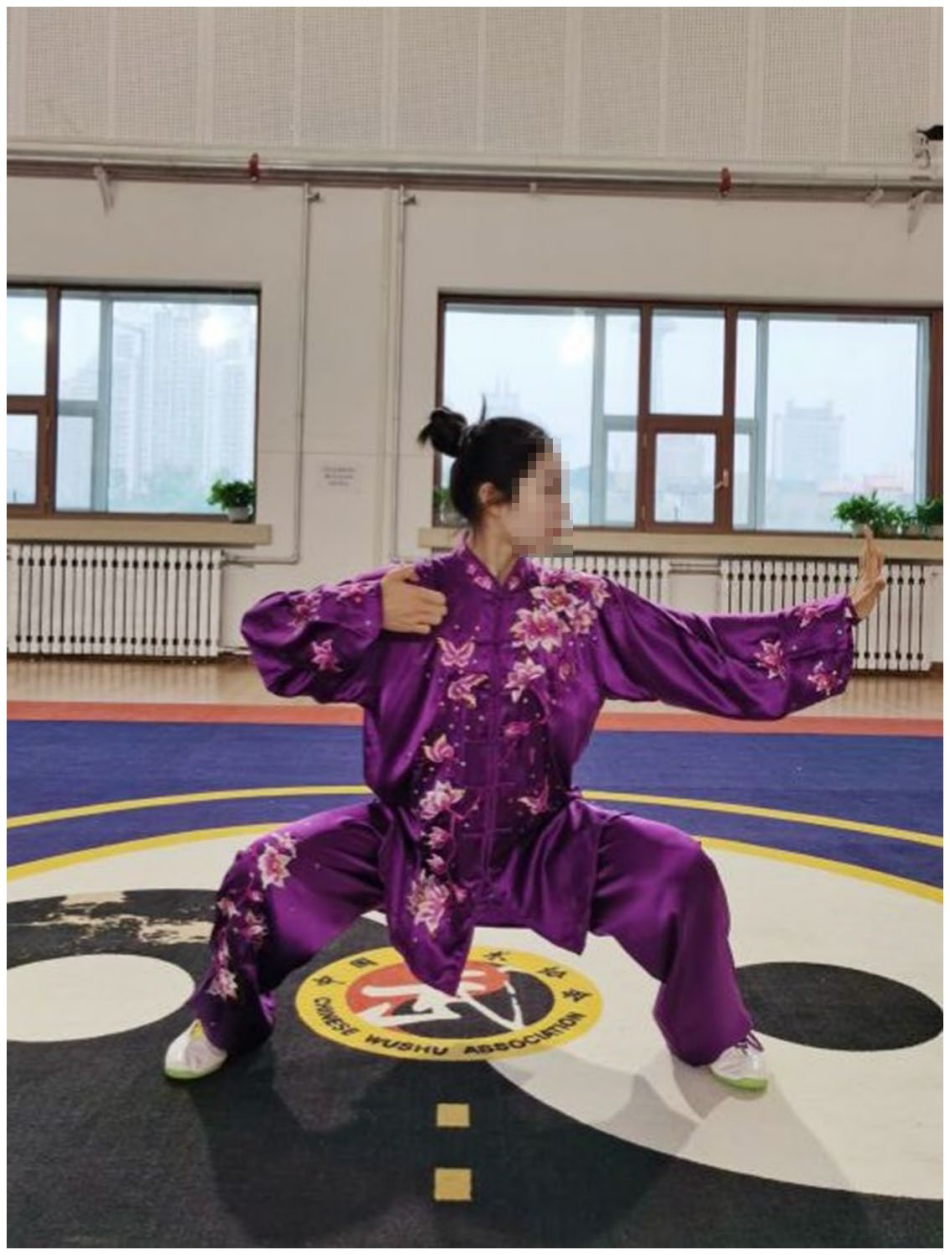
Chen Jian Zhui Zhou (drop shoulders and sink elbows) in the movement Zuo You Kai Gong Si She Diao (posing as an archer shooting both left- and right-handed).

**Figure 3:**
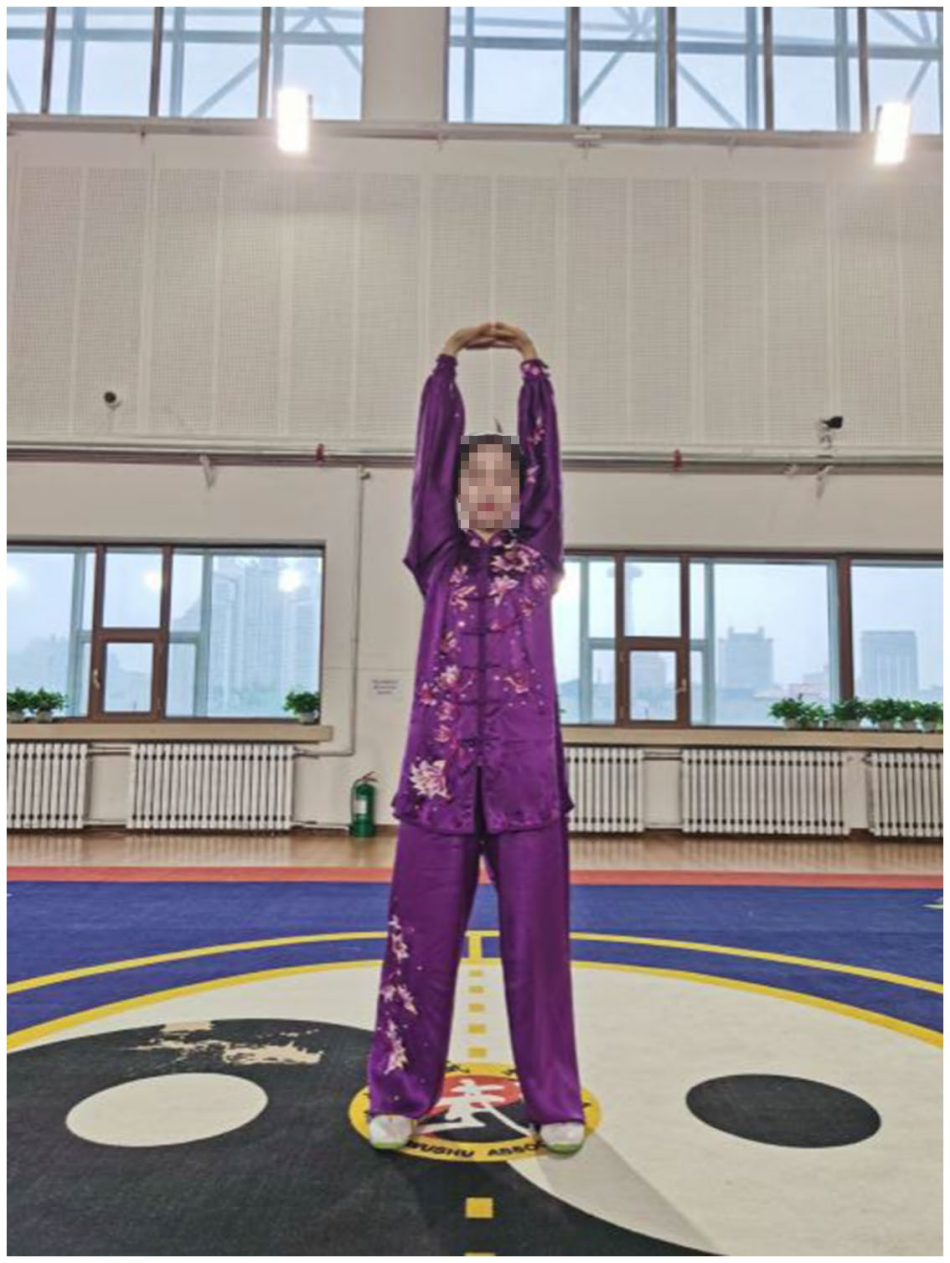
Liang Shou Tuo Tian Li San Jiao (holding the hands high with palms up to regulate the internal organs).

After an 8-week intervention, data relating to upper extremity muscles were collected for sEMG in the current study. The findings showed that patients who practiced the Qigong exercise experienced a significant decrease in CCR of upper limb muscles compared with the control group. This suggested that Qigong exercise positively reduced muscle spasms in stroke patients. In Qigong exercises, the core movements of the upper limbs are “Chen Jian Zhui Zhou (Drop Shoulders and Sink Elbows).” It can be demonstrated vividly in Figure 2. The movement in Figure 2 is “Zuo You Kai Gong Si She Diao (Posing as an Archer Shooting Both Left- and Right-Handed),” which requires the subjects to lower down the shoulders and elbows when they are shooting. In Starting movement, two hands become a ball posture, require sink the shoulders and drop the elbows, and keep a hollow space under the armpits. Both hands do upward and downward can minimize shoulder shrugs, keep shoulders and elbows relaxed, and to some extent, loosen the joints and the surrounding ligaments (Gao et al., 2014). In the action of Figure 3, the shoulder muscles force downward to prevent the shoulder from lifting, then drop the hands while relaxing the shoulders, elbows and chest. Therefore, with the joint tendons relaxed and tensed, when maintaining upper limb movement, the patients only need to overcome their own gravity without using additional force. Since too much exertion of antagonistic muscles would result in spasms in stroke patients (Becker et al., 2019), it is highly probable that Qigong exercises can relieve the exertion of antagonist muscles by making patients relax their upper limbs as much as possible.

**Figure 4:**
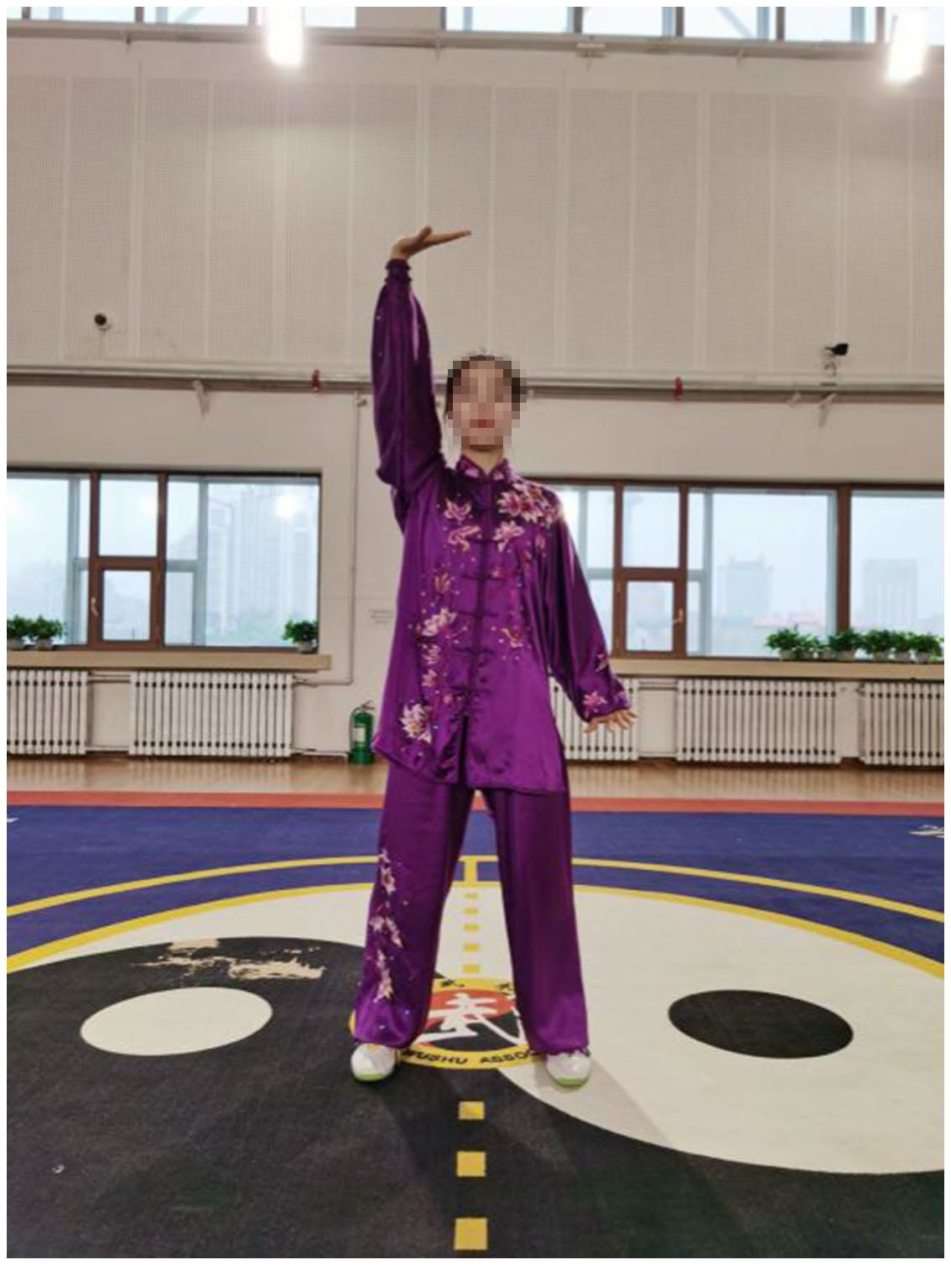
The right palm is pushed up and the left palm is pressed down.

**Figure 5:**
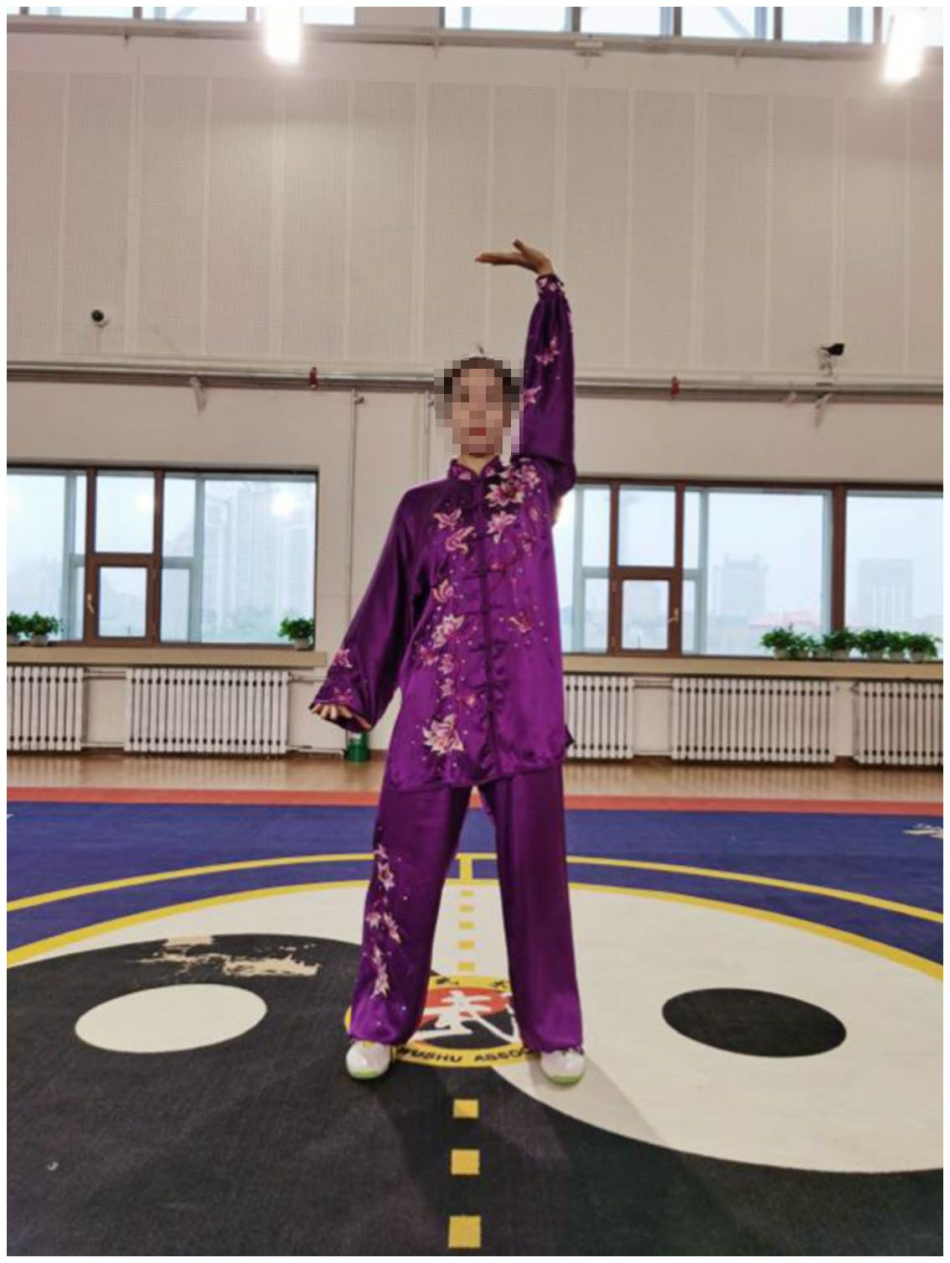
The left palm is pushed up and the right palm is pressed down.

In Qigong exercises, agonist muscle are properly exerted and antagonist muscles are kept at a corresponding degree of relaxation, which is conducive to unblocking the meridians, slipping the joints and strengthening the muscles and bones. The upper limbs are flexed at the elbows, which not only ensures smooth movement of the skeletal muscles and joints, but also maintains stability and improves muscle strength. In the qigong movements, the upper limbs all have elbow flexion movements. For example, the elbows are bent and risen in the first movement (Figure 3); stretched and extended sideways in the second movement (Figure 2). Moreover, the palms are pushed up and pressed down alternately with the elbows bent in the third movement (Figures 4, 5). Three muscle movements are mainly used in this process, including eccentric contraction, concentric contraction, and isokinetic contraction (Ge et al., 2004) with the coordination of agonistic muscles and antagonistic muscles (Christou et al., 2003). Stroke patients usually have high CCR accompanied by central nervous system damage, which directly leads to abnormal changes in coordinated muscle contraction (Desrochers et al., 2016). Qigong exercises require slow and even, coherent and gentle, loose and tight combination, breathing and consciousness together. Patients can perceive the internal and external changes of the limbs, and thus stimulate and improve the central nervous system. In this way, Qigong exercises may promote the coordination and exertion of agonistic and antagonistic muscles in stroke patients.

**Figure 6:**
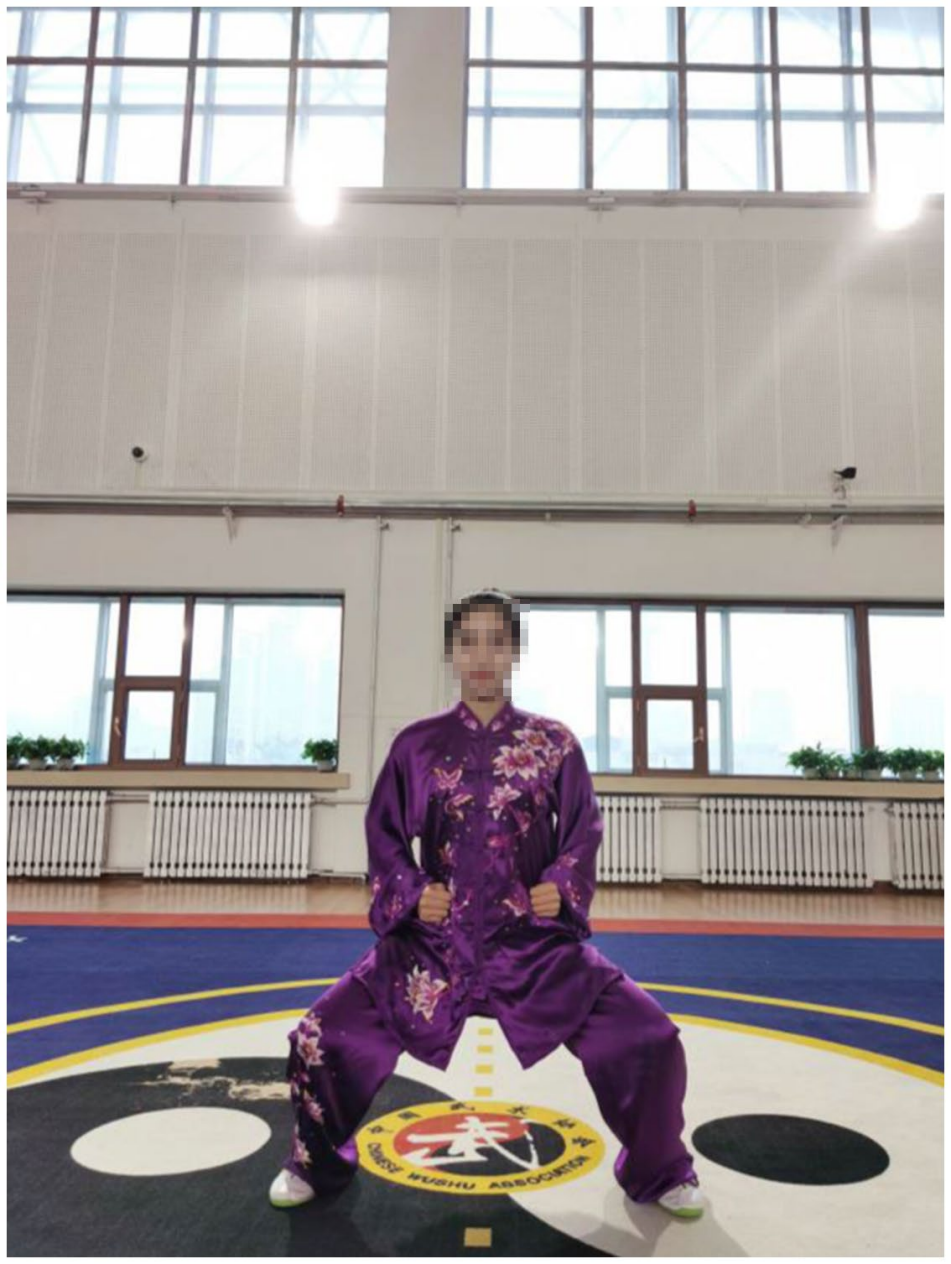
Horse stance.

The transformations, which smooth out the gaps between the movements, are primarily Horse-stance (Figure 6) and Standing-stance based. It requires the exercisers to bend their knees slightly, take a step sideways to either the left or the right with single foot, and withdraw the step back. The gravity center of the body shifts from either foot to the place between the feet. In the lower limb movement transformation, the lower limb muscle strength is exercised, and the balance ability and stability of the lower limbs are improved. In the open-eye balance test, the Qigong group performed significantly better after 8 weeks, especially in Y dev., Y speed index, and Area index, which indicated that Qigong exercise improved the balance of the stroke patients. However, the Qigong group did not differ from the control group in the closed-eye test. It can be seen that in the absence of sensory assistance, the Qigong group had no improvement in performance in the left, right, front or back directions. Unlike in the opened-eye test, the maintenance of static posture depends more on attention than dynamic posture change. This may also be the main reason why there is no significant change in the center of gravity micro-control index in stroke patients without conscious assistance.

**Figure 7:**
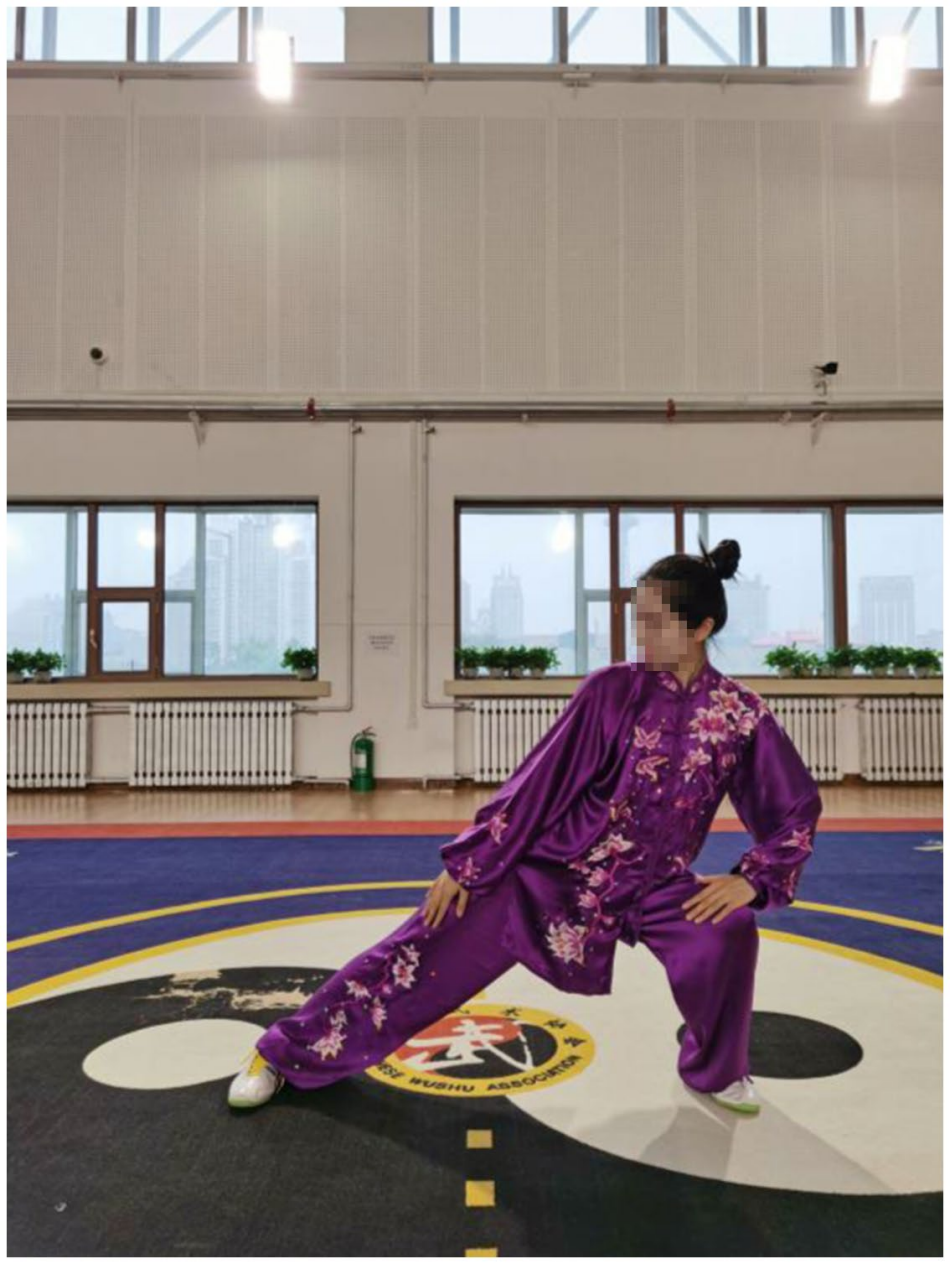
The movement of gravity center to either the left or the right from Yao Tou Bai Wei Qu Xin Huo (swinging the head and lowering the body to relieve stress).

**Figure 8:**
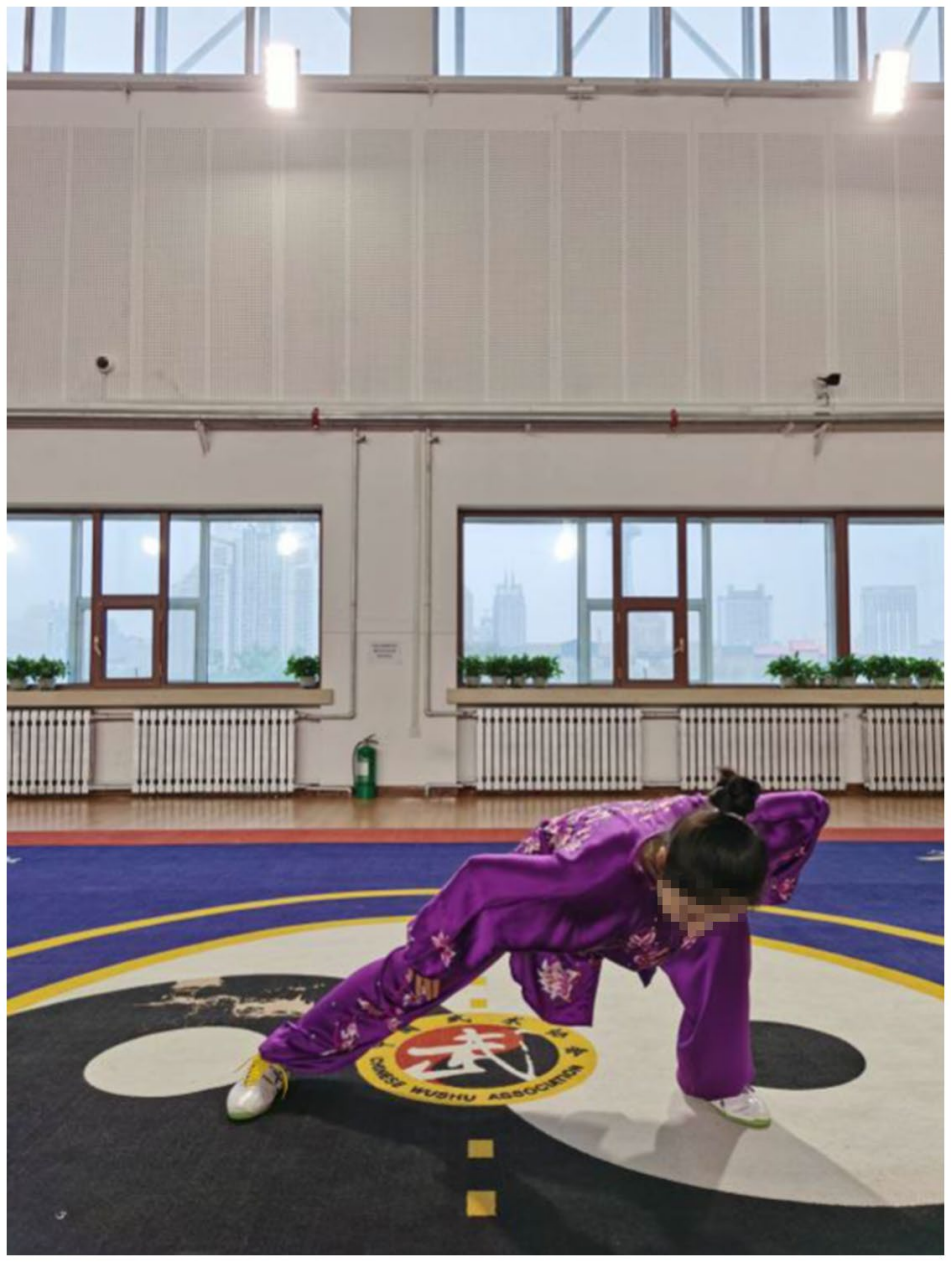
The movement of gravity center to either the front and the back from Yao Tou Bai Wei Qu Xin Huo (swinging the head and lowering the body to relieve stress).

**Figure 9:**
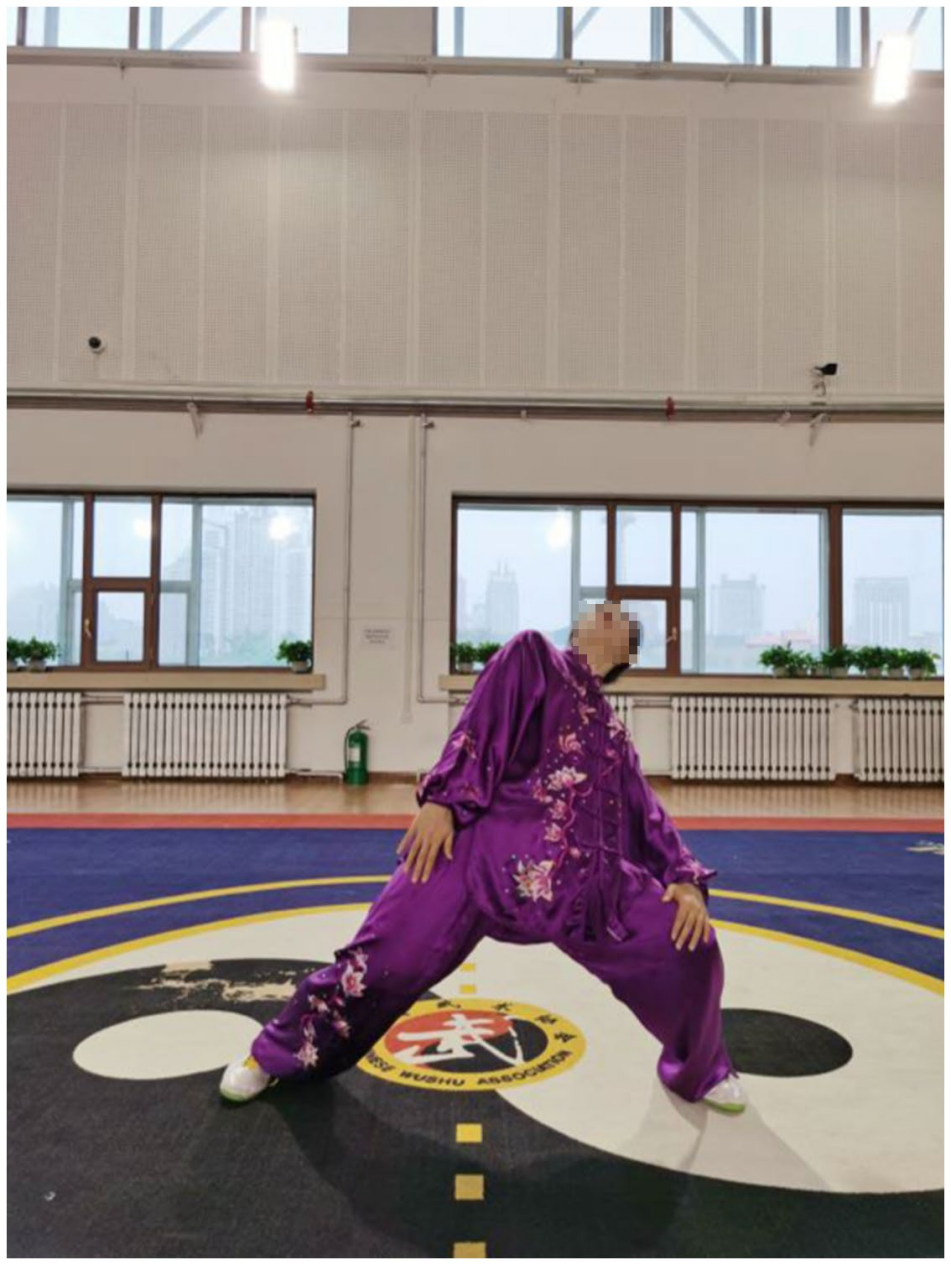
The movement of gravity center to either the up and the down from Yao Tou Bai Wei Qu Xin Huo (swinging the head and lowering the body to relieve stress).

**Figure 10:**
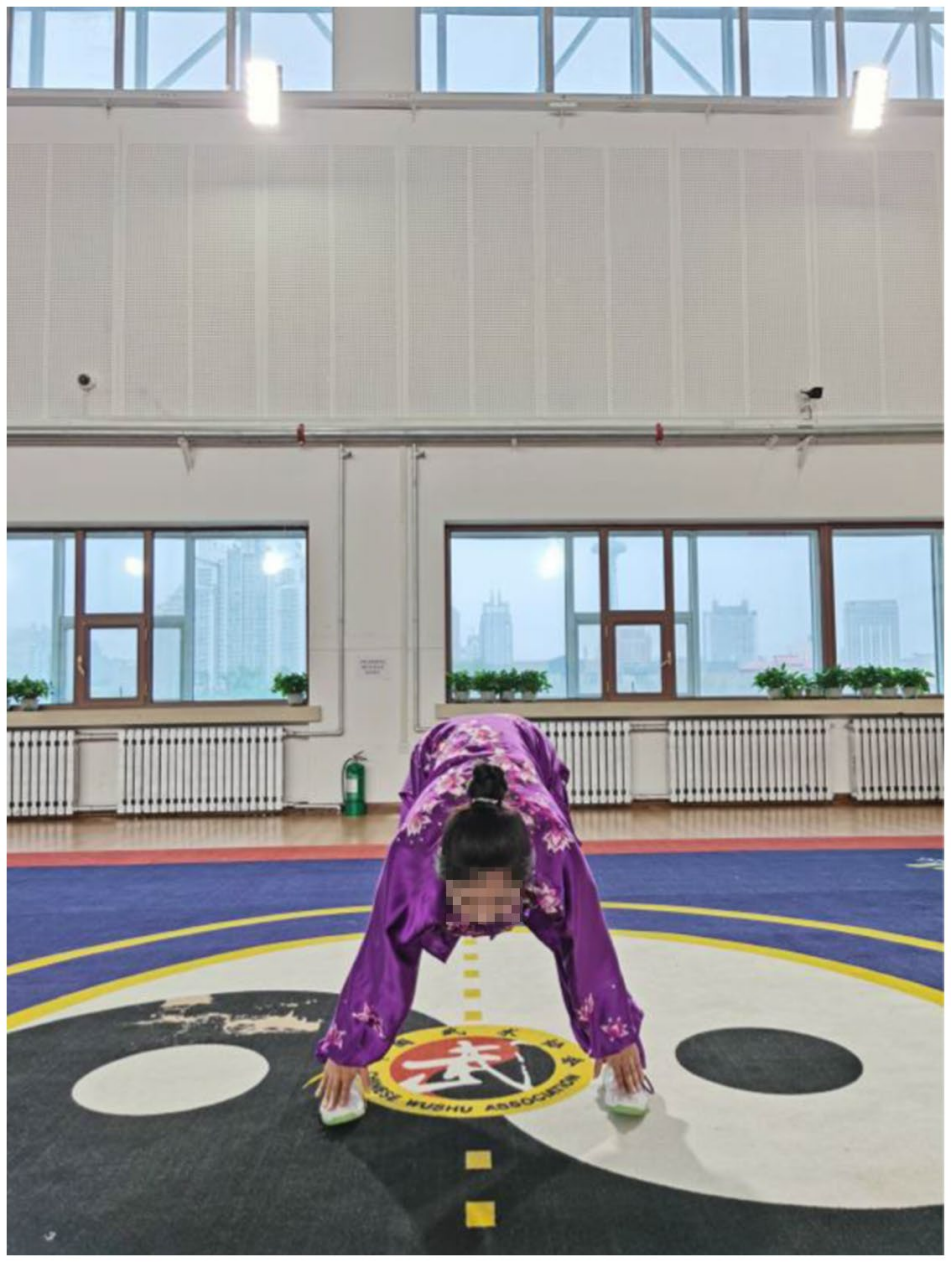
Liang Shou Pan Zu Gu Shen Yao (moving the hands down the back and legs and touching the feet to strengthen the kidneys).

The decrease of muscle strength in stroke patients is one of the main causes of motor function damage, including balance. The center of gravity is one of the most important factors affecting balance. The big toe and the first metatarsal bone also play a major role in adjusting bodily posture (Meyer et al., 2004). Qigong exercise involves movements in six directions, front and back, left and right, up and down. The gravity center of the body can be either in the up, down, left and right directions. The basic technical movements of Qigong require that the rotation of upper body and the transfer of gravity center should be completed during the horse stance and standing stance, especially when the exercisers bend their knees and squat down. For example, in the fifth movement (Figures 7–9), exercisers should rotate the upper body in a circle and transfer the gravity center from the left to the right, or from the right to the left, when shifting the Horse stance to either one side Horse stance. In the sixth movement (Figure 10), exercisers extend their legs fully first. When lifting the arms above the head, exercisers begin to rise the upper body with the arms, thus the upper body recurves and both the legs and arms can be fully stretched.

Previous studies found that during Qigong practice, the maximum pressure on the sole of the foot is mainly concentrated on the first metatarsal bone and the big toe, while in normal walking it is more concentrated on the second, third, fourth, and fifth areas (Mao et al., 2006). The results in the present study showed that the Y dev. and Y speed index of the Qigong group was significantly lower than that of the control group. Therefore, Qigong exercises effectively improved the balance of the center of gravity in stroke patients.

Quality of life was assessed in terms of physical health, psychological state, social relationships, environment, and a total score. Qigong is a whole-body exercise, which is a mind–body exercise that harmonizes body, breath and mind. When practicing Qigong, the consciousness is dominant and the breathing is coordinated with the movements. Each movement has a breathing method to match with. For example, exercisers inhale when rising the arms and exhale when putting down the arms in the first movement; inhale when turning the hands into the palms and exhale when stretching sideways in the second movement; inhale when rotating the arms outward, exhale when withdrawing the arms back, and hold the breath for a second when keeping the posture still in the fourth movement.

The results indicated that the score for physical health in the Qigong group improved significantly while psychological health also improved significantly by the end of the 8 weeks, similar to the results of earlier studies. In a 6-month Baduanjin exercise program for breast cancer survivors, there were significant improvements in heart rate variability, shoulder range of motion on the affected side, depression, and quality of life (Ying et al., 2019). Furthermore, paced breathing synchronized with rhythmic muscle contraction leads to more resilient activation of the parasympathetic response than either alternating contractions or breathing alone, which may help explain the benefits of mind–body disciplines (Chin and Kales, 2019). The number of Qigong movements is small, and the route is not complicated, easy to learn. Practitioners can adjust the intensity and difficulty of the movements according to their own physical conditions. Before the experiment, most of the stroke patients were afraid of taking up Qigong exercise due to their limitations in physical activity. However, after being exposed to Qigong exercises, they were able to complete the basic movements, which may explain why the Qigong group had higher scores for their psychological state than the control group after 8 weeks and why as the study progressed, the psychological state of stroke patients improved.

In our study, the control group showed some improvements but were less marked than the Qigong group in the same amount of time. Although there was no significance between the groups, the Qigong group performed better than the control group after 8 weeks, which has been shown in some of the data comparisons except for the significant ones. For example, Qigong group has made greater progress in the TL of static balance in the open-eye of biped in Table 3, the Y speed of static balance in the closed-eye of biped in Table 4, the Social relationships of WHOQOL-Bref in Table 5.

Regarding the insignificant results between groups, we have also revised our experimental design and put it down to three reasons. First, an 8-week intervention was not enough for more significant effects. Second, the results was not that noticeable with low frequency of Qigong exercise, 3 times a week in this experiment. Third, the time spent on Qigong exercise each time should be longer. We would like to observe the effect of Qigong exercise on stroke patients and obtain the optimal scheme by increasing the length, frequency and time in the future design and experiment.

## 5. Limitations

There are some limitations in this study that should be acknowledged. First, the characteristics of stroke patients were mixed (such as different levels of impairment and types of impairment), which makes it difficult to generalize from the results. Second, the exclusion criteria were very strict and nearly 23% of stroke patients had anxiety and depression, which led to the small sample size. Third, the duration of the intervention was 8 weeks, which was not enough for the follow-up assessments to examine the long-term effects of the intervention. It will be better if we could increase the length, frequency and time of Qigong intervention. Fourth, it was difficult to ensure effective blinding in this study. Fifth, we have not done the tests of National Institute of Health stroke scale (NIHSS) and motor compromise.

## 6. Conclusion

After 8 weeks of intervention, Qigong exercises can effectively improve the upper extremity muscle activity, balance, and quality of life in stroke patients. Qigong exercise not only improves balance but the coordination and exertion of antagonistic and active muscles in stroke patients, and quality of life. The psychological state of the Qigong group was also significantly improved in this study but given the small sample size, further studies are needed to provide stronger evidence with larger samples. In addition, multivariate analyses may be applied to understanding the relationship between hypertension, diabetes, obesity, hyperlipidemia, non-sudden stroke, internal capsule involvement, pontine morphology, and other factors, and the motor dysfunction associated with stroke.

## Data availability statement

The original contributions presented in the study are included in the article/supplementary material, further inquiries can be directed to the corresponding authors.

## Ethics statement

The studies involving human participants were reviewed and approved by the Ethics Committee of the Second Affiliated Hospital of Heilongjiang University of Chinese Medicine. The patients/participants provided their written informed consent to participate in this study. Written informed consent was obtained from the individual(s) for the publication of any potentially identifiable images or data included in this article.

## Author contributions

HY, BL, ZZ, and XL: conceptualization. HY, BL, LF, and XL: methodology. HY and LF: software. HY, ZZ, and XL: validation. HY and XL: formal analysis, writing—original draft preparation, and writing—review and editing. HY, BL, and LF: investigation and data curation. HY, ZZ, BL, and LF: resources. XL: supervision. HY: project administration and funding acquisition. All authors have read and agreed to the published version of the manuscript and made substantial contributions to conception and design.

## Funding

This research was funded by the Natural Science Foundation of Heilongjiang Province (No. LH2020H009).

## Conflict of interest

The authors declare that the research was conducted in the absence of any commercial or financial relationships that could be construed as a potential conflict of interest.

## Publisher’s note

All claims expressed in this article are solely those of the authors and do not necessarily represent those of their affiliated organizations, or those of the publisher, the editors and the reviewers. Any product that may be evaluated in this article, or claim that may be made by its manufacturer, is not guaranteed or endorsed by the publisher.
